# Adaptation of the inferior temporal neurons and efficient visual processing

**DOI:** 10.3389/fnbeh.2024.1398874

**Published:** 2024-07-26

**Authors:** Yukako Yamane

**Affiliations:** Neural Computation Unit, Okinawa Institute of Science and Technology Graduate University, Okinawa, Japan

**Keywords:** repetition suppression, adaptation, efficient coding, predictive coding, inferior temporal (IT) cortex

## Abstract

Numerous studies examining the responses of individual neurons in the inferior temporal (IT) cortex have revealed their characteristics such as two-dimensional or three-dimensional shape tuning, objects, or category selectivity. While these basic selectivities have been studied assuming that their response to stimuli is relatively stable, physiological experiments have revealed that the responsiveness of IT neurons also depends on visual experience. The activity changes of IT neurons occur over various time ranges; among these, repetition suppression (RS), in particular, is robustly observed in IT neurons without any behavioral or task constraints. I observed a similar phenomenon in the ventral visual neurons in macaque monkeys while they engaged in free viewing and actively fixated on one consistent object multiple times. This observation indicates that the phenomenon also occurs in natural situations during which the subject actively views stimuli without forced fixation, suggesting that this phenomenon is an everyday occurrence and widespread across regions of the visual system, making it a default process for visual neurons. Such short-term activity modulation may be a key to understanding the visual system; however, the circuit mechanism and the biological significance of RS remain unclear. Thus, in this review, I summarize the observed modulation types in IT neurons and the known properties of RS. Subsequently, I discuss adaptation in vision, including concepts such as efficient and predictive coding, as well as the relationship between adaptation and psychophysical aftereffects. Finally, I discuss some conceptual implications of this phenomenon as well as the circuit mechanisms and the models that may explain adaptation as a fundamental aspect of visual processing.

## 1 Introduction

The inferior temporal (IT) cortex occupies a later stage in the ventral visual pathway and is responsible for object recognition. Compared to the lower visual areas such as the primary or secondary visual areas (V1 and V2) which have small receptive fields and relatively faithful responses to low-level image features or intensity, neurons in the IT cortex are characterized by their large receptive field, sensitivity to complex shapes, object images or categories, and invariance to substantial changes in the pixel values caused by resizing, rotation, or shading (Ito et al., 1995; Logothetis et al., 1995). Output from the IT neurons innervates various areas such as the rhinal cortex, amygdala, striatum, and prefrontal cortex (Kravitz et al., 2013). Thus, throughout the ventral visual pathway, information dealt within the corresponding area seems to drastically change from the one strongly related to the outside environment and input image statistics to the one advantageous for understanding the surrounding environment, memorizing, associating important items with values, or performing tasks that may have considerable consistency with our thoughts and languages.

Numerous studies examining the responses of individual IT neurons have revealed interesting characteristics such as two- or three-dimensional shape tunings (Op de Beeck et al., 2001; Brincat and Connor, 2004; Yamane et al., 2008; Hung et al., 2012), objects or their category selectivity (Hung et al., 2005; Kiani et al., 2007), as well as faces (Gross, 2008) or scenes (Vaziri et al., 2014). While these basic selectivities have been studied assuming that their response to the stimuli is relatively stable (Op de Beeck et al., 2001; Srihasam et al., 2014), physiological experiments have revealed that the responsiveness of IT neurons is also dependent on visual experience (Tovee et al., 1996; Kobatake et al., 1998; Li and DiCarlo, 2010; Meyer and Olson, 2011; Woloszyn and Sheinberg, 2012; Vogels, 2016). The time required to evoke changes in responsiveness ranges from within 1 s such as repeated presentations to months or years of exposure. Some of these changes occur without task demands such as remembering or attending to the image; however, simply viewing the object stimuli in a particular order or frequency is sufficient to evoke changes. There are questions regarding what significance those changes hold for visual processing, how they impact coding by population neurons, and how a dynamic response can be compatible with stable coding. In the following sections, I summarize various modulation types observed in the IT neurons. Among these, I particularly focuse on repetition suppression (RS), where the responses of IT neurons to stimuli that are the same or similar to the preceding stimulus are attenuated (Desimone, 1996). The more widespread word, sensory adaptation, is also used in other visual areas. Although adaptation and RS are often used interchangeably, especially in visual processing in the ventral stream, I will refer to the (mainly suppressive) modulation of IT neurons as RS and modulation of other areas as adaptation here. I discuss the properties of RS and introduce a study on adaptation in the lower visual areas and its implications. Additionally, the discussion extends to aftereffects, context modulation, and efficient coding. I argue that the RS and adaptation are closely related to visual information processing, and finally, discuss some computational models including efficient coding and excitation/inhibition (E/I) balanced networks, that possibly connect hierarchical visual processing as inference and adaptation.

## 2 Types of changes of the IT neural activity by experience

### 2.1 Task-driven modulations

Several studies have shown that experience modulates the activity of the IT neurons. Task-dependent and -independent modulations and changes in activity have been reported. In the research on task-dependent modulation, the underlying concept revolves around examining how feature selectivity of the IT neurons is influenced by learning in adults achieving proficiency in tasks such as discrimination and categorization. For instance, Kobatake et al. (1998) subjected monkeys to a shape discrimination task, comparing the responses of the IT neurons to stimuli used in the discrimination task and those not used in the task. Neurons exhibiting strong responses to stimuli used in the discrimination task were more prevalent in the trained monkeys. In this experiment, animals were anesthetized during neural recording, eliminating considerations for factors such as attention or reward influences. Moreover, in experiments involving a categorization task (Sigala and Logothetis, 2002), visual features crucial for categorization were reported to exhibit sharper selectivity than irrelevant features after learning. These studies suggest that the representations of visual features relevant to the task undergo selective changes during month-long training sessions. Additionally, the IT neurons modulate their responses through association learning. The learned association between two stimuli enables the IT neurons to respond to the pair of associated stimuli through collaboration with the memory system, as revealed in a series of studies (Miyashita and Hayashi, 2000; Miyashita, 2004; Hirabayashi and Miyashita, 2014).

### 2.2 Long-term visual experience causes modulations

In addition to task-driven modulation, changes solely caused by visual experience have been widely reported. For instance, after months of passive exposure to specific stimuli without any task demanding memory or discrimination, IT neuronal responses differed between familiar (exposed) and novel stimuli in animals (Anderson et al., 2008; Woloszyn and Sheinberg., 2012). Woloszyn et al. demonstrated that for familiar stimuli, responses in the majority of neurons decreased; however, within them, the selectivity of putative excitatory neurons responding to familiar stimuli increased. Furthermore, in addition to stimuli familiarity, passive viewing with a consistent stimuli sequence (in which one specific stimulus is always followed by another, whereas the order of other stimuli is randomly shuffled) can implicitly be associated with stimuli (statistical learning), inducing changes in the IT neuronal activity (Meyer and Olson, 2011; Meyer et al., 2014; Ramachandran et al., 2016, 2017; Kaposvari et al., 2018; Esmailpour et al., 2023). These studies revealed that expected stimuli led to a decrease in the firing rate and violating the presentation order of the stimuli led to an increase in the firing rates, indicating compatibility with predictive coding (Rao and Ballard, 1999). The relationship between expectation-related modulation and predictive coding has been the focus of much attention, leading to various experimental investigations across different task paradigms (Feuerriegel et al., 2021). The concept of “predictive coding” is revisited later in this manuscript.

Thus, presenting stimuli with controlled statistical properties for relatively extended periods such as weeks or months can modulate IT responses with the consistent stimuli sequence. Notably, IT neurons do not distinguish triplets (the order of three consecutive stimuli) or higher orders even though they are referred to as “sequences” (Meyer et al., 2014). This emphasizes the importance of temporal proximity in inducing changes in the IT neurons. Indeed, temporal proximity is a characteristic of the usual input to the visual system (i.e., temporal continuity: objects do not abruptly appear or disappear), and it is conceivable that such statistical properties of the external world influence object representations in the IT cortex. Stimuli repeatedly presented with temporal closeness have been demonstrated to produce more similar representations in single neurons (Li and DiCarlo, 2010; Jia et al., 2021).

### 2.3 Short-term modulation

IT neuronal activity can be modulated without long-term exposure. Attention can increase the activity of single IT neurons (Moran and Desimone, 1985) and change the population representation of objects (Sereno and Lehky, 2018). Sereno et al. examined the discriminability of stimulus shape identity by IT neuronal population when recorded while the subject is attending to shape or to location. They found that the discriminability is higher during attention to shape. The effect of attention is thought to originate from the frontal cortex (Ramezanpour and Fallah, 2022), and the IT cortex is suggested to be involved in an object-based attention network. RS is another modulation type. Responses to stimuli that are the same or similar to the immediately preceding stimulus are attenuated. The RS has been extensively studied since it was reported [Response reduction by stimulus repetition was first reported by Brown et al. (1987) and Baylis and Rolls (1987). The name ‘RS’ was used in a later paper (Desimone, 1996)]. Initially, it was suggested that the IT neurons may be the filter to pass new, unexpected stimuli (Miller et al., 1991), or that RS may be important to discriminate novel objects (Ringo, 1996), or that RS is a neural correlate of the psychological phenomenon of priming in which once an image is presented, psychophysical discriminability is increased (Wiggs and Martin, 1998). The phenomenon where the neural response to the same stimulus changes is generally called sensory adaptation, and it is common across different sensory areas [ex., olfactory bulb (Scott, 1977), barrel cortex (Khatri et al., 2004), and auditory cortex (Ulanovsky et al., 2003)]. Adaptation-like modulation has been observed in the visual neurons of lower and higher orders, including V1 (Müller et al., 1999; Patterson et al., 2013), V4 (Wang et al., 2011), MT (Kohn and Movshon, 2004; Patterson et al., 2014), MST (Price and Born, 2013), and IT (Baylis and Rolls, 1987; Brown et al., 1987). Some psychological phenomena have been associated with visual neuronal adaptations, and various mechanisms have been proposed. Despite the relatively large amount of literature and observation throughout ventral visual areas, its significance in visual processing is yet to be completely elucidated. The following sections will provide various discussions regarding the properties of RS in IT and adaptation in other visual areas and topics related to adaptation in the visual system, including the possible interpretation and mechanism of adaptation.

## 3 Properties of repetition suppression in IT neurons

### 3.1 General property

While reduction of response to a similar stimulus is observed throughout the ventral visual pathway, it appears to be most prominent in the IT neurons when compared using the same stimuli across areas (Yamane et al., 2023). An interesting property of this suppression in IT is that the extent of the suppression induced by repeated stimuli depends on the combination of the stimulus used and the recorded neuron. In other words, even if two different stimuli evoke similar response strengths, the magnitude of the RS effect (suppression) induced by the two stimuli differs for each neuron (Sawamura et al., 2006; Liu et al., 2009; De Baene and Vogels, 2010). Additionally, RS can be induced invariantly in position or size (De Baene and Vogels, 2010), demonstrating similarity to general stimulus selectivity in individual IT neurons. The reduction of the response cannot be explained by attention reduction. De Baene and Vogels (2010) compared RS during an attention-demanding task and a passive fixation task to explore the interaction between attention and RS in IT. The results showed that the strength of RS did not differ between the attended and non-attended conditions. This lack of difference suggests that there is likely no strong interaction between attention and RS. This finding contrasts with expectation suppression discussed in the later section, which often requires attention. RS-like suppression occurs even during free viewing. Repeated fixations on the images of the same object evoke suppression of the response of IT neurons (Yamane et al., 2023). Therefore, it is a phenomenon that occurs both under limited (fixation task) conditions and under relatively free conditions that allow free viewing.

RS is closely related to the stimulus selectivity of individual IT neurons and cannot be merely explained by stimulus-nonspecific neuronal fatigue. Following this notion, a comparison of IT excitability before and after direct (without using visual stimuli) photostimulation of IT neurons demonstrated sufficient responses even after photostimulation, contradicting the theory of fatigue in IT neurons themselves (Fabbrini et al., 2019). Thus, changes in input (which may separately vary with each stimulus) such as synaptic depression are suggested to be involved in stimulus-dependent RS (Vogels, 2016). Examining the relationship between neuronal selectivity and suppression in the IT neurons is extremely challenging, primarily because of the multidimensional and complex nature of selectivity in the IT neurons.

### 3.2 Adaptation of dorsal stream neurons

In the middle temporal (MT) area, adaptation has been studied using moving gratings, and it has been reported that motion direction tuning becomes sharper due to adaptation (Kohn and Movshon, 2004). For such simple stimuli, it has been shown that inherited input from V1 plays a significant role (Kohn and Movshon, 2003). Adaptation to more complex stimuli, such as plaids, can also be explained by pooling the changes in V1 input (Patterson et al., 2014). However, in the case of dot stimuli, there is a report that direction tuning does not change after adaptation, but speed preference does (Yang and Lisberger, 2009). Therefore, the type of stimulus can be an important factor. However, in general, the selectivity of MT cells is easier to parameterize than that of IT cells, making it easier to examine the effects of adaptation to the tuning. In addition, a notable feature of adaptation in MT is that there are a significant number of cells that are not suppressed but enhanced. This enhancement occurs because MT cells are enhanced by motion stimuli in the direction opposite to that of the adaptors’ motion. Such a feature is not observed in RS in IT. This difference is thought to be due to the distinct computations performed in each area—motion direction extraction in MT and complex feature extraction in IT (Kar and Krekelberg, 2016).

## 4 Studies in the lower visual areas and their implication in the RS of IT neurons

Insights into the nature, mechanism, and implication of the visual computation of adaptation in the lower visual neurons with relatively (though not absolutely) simple stimulus selectivity and known circuits may help understand and allow for specification of the RS mechanism and impact on the population activity of the IT neurons. I hypothesize that the underlying principle of the adaptation in IT is similar to the lower visual area and the empirical difference related to RS roots from the computations performed by the cell populations in both areas (e.g., contrast extraction or complex shapes). In V1, adaptation is discussed as the short-term modulation of neural activity influenced by previous stimuli; this includes suppression as well as enhancement.

Relatively few reports have examined adaptation via electrophysiological experiments in the intermediate areas of the ventral visual pathway such as visual areas V2 and V4 neurons (Tolias et al., 2005; Crowder et al., 2006; Wang et al., 2011). However, a considerable amount of literature is available on the adaptation in the V1 neurons (see review; Kohn, 2007). I focus on some intriguing topics in the experimental and theoretical fields concerning V1 neuronal adaptation and attempt to extend the discussion to RS in the IT neurons. Repetition of the same or similar stimuli can be considered as the repeated presentation of redundant visual input, and reducing this redundancy in the neural code aligns naturally with efficient coding (Attneave, 1954; Barlow, 1961; Wainwright, 1999), where sensory circuits encode maximal information about their inputs, reducing redundancy. Here, I explore the discussion related to information maximization conveyed by neurons through redundancy reduction and consider experimental results that support and argue for this notion. Additionally, the discussion includes the tilt aftereffect, a well-known psychological phenomenon, and its neuroscientific underpinnings. These topics are interrelated.

### 4.1 Efficient coding and adaptation

The concept of efficient coding has evolved since it was first proposed in the field of neuroscience (Attneave, 1954; Barlow, 1961) and has been used to explain various experimental results (Simoncelli and Olshausen, 2001; Chalk et al., 2018; Price and Gavornik, 2022). In vision specifically, efficient coding often refers to reducing redundant information and maximizing the information content when visual images are coded. For instance, contrast adaptation is known to exist in the retina and V1 neurons, which involves the strength of the response adaptively changes based on the history of past stimulus contrasts (mean and std), thereby maximizing the information conveyed in the current stimulus distribution. Such adaptive changes are observed in the motion-selective neurons of flies (Brenner et al., 2000; Fairhall et al., 2001) and the auditory cortex of songbirds (Nagel and Doupe, 2006), suggesting a mechanism shared among different animals and across different sensory modalities. However, adaptation extends beyond these primary statistical measures even at the early visual stages. In the retinas of salamanders and rabbits, different adaptations occur for stimuli with the same contrast and luminance but with different stimulus patterns (Hosoya et al., 2005). In V1 neurons, receptive fields (RF) have been demonstrated to differently adapt to artificial (noise images) and natural images to maximize the information content that is specific to adapted images (Sharpee et al., 2006). In their study, the visual system adjusted the output of the V1 neurons to efficiently account for the characteristics of natural images, where low-frequency components had a higher probability of occurrence.

The aforementioned examples reveal that given its limited resources, the neural system can adapt to maximize the information relevant to the processing stage. The importance of the lower visual features such as contrast, orientation, and frequency in areas such as the retina and V1 neurons is unquestionably important. However, confirming whether information is processed more efficiently becomes more complex as the visual hierarchy ascends. We must consider the relevance of the information in the processing stage, which is directly related to neural coding. In cases higher than V1, what specific information the neurons are attentive to or extracting is not always clear.

Nonetheless, psychophysical experiments have provided the statistical feature that is important for perceptual judgment and guided the analysis of efficient coding in V2. Hermundstad et al. (2014) carefully identified the statistical properties of natural images and identified those most variable and thus the least predictable and, therefore, the most informative statistical feature. Based on the identified features, Yu et al. (2015) examined the type of efficient coding at V2. In subsequent recordings of V1 and V2 neurons from macaque monkeys, they demonstrated the emergence of the response dependency in V2 neurons on the identified informative feature. This result indicates that the constraint on efficiency in V2 originates from input sampling— in other words, the relevance of information of input to behavior— not output capacity, as the original efficient coding suggested. Thus, the result demonstrates that different types of efficiency are optimized in different areas.

In addition to the difficulty in identifying relevant information, a population’s amount of encodable information can vary depending on the strength of correlations between the cells in the population (Moreno-Bote et al., 2014; Shamir, 2014). Therefore, concluding whether changes due to adaptation in single cell response make population coding more efficient is not straightforward. Simulations of the V1 population indicate that coding accuracy can increase or decrease depending on the adaptation mechanism and stimulus (Cortes et al., 2012). Consequently, whether the information coded by the population is genuinely improved and becomes efficient through adaptation, even in V1, remains unclear.

In addition to the challenge mentioned earlier in evaluating efficiency, several potential conflicting issues exist that may prevent encoding efficiency such as limitation of resources or sampling, or, more importantly, constraints from decordability. Even though highly efficient encoding is possible, it can be challenging to decode in downstream areas and thus can be not helpful for behavior (Tesileanu et al., 2022). Furthermore, the behaviors in which the ventral visual system is engaged may be diverse. Task information reformatting, rather than maximization (Gaspar et al., 2019), is also an essential direction that needs to be considered, especially when considering coding efficiency in the IT cortex.

### 4.2 Aftereffects and adaptation

The tilt aftereffect is a well-studied intriguing psychophysical adaptation phenomenon (Gibson and Radner, 1937). It refers to a systematic bias in the perception of the orientation after exposure to a grating with a particular orientation. The difference between adaptor and test stimulus orientations determines the perception of tilt direction. Interestingly, aftereffects occur also with more complex stimuli. For example, the difference in the three-dimensional viewpoint of a face image evokes bias in viewpoint (Fang and He, 2005). Some properties of face angle aftereffect are similar to the tilt aftereffect in oriented gratings. For example, the angular tuning function of the face viewpoint aftereffect is similar to the angular tuning function of the tilt aftereffect (Chen et al., 2010), indicating a common process across extensive regions of the visual pathway, including the IT cortex (Leopold et al., 2001).

One interesting hypothesis suggests that the mechanism (or principle) underlying the occurrence of the tilt aftereffect and the tilt illusion may be the same (Schwartz et al., 2007; Clifford, 2014). In tilt illusion, spatially separated adapters and stimuli are presented simultaneously. The oriented grating shown in the center is perceived as tilted in the opposite direction to the orientation of the surround. Tilt aftereffects and tilt illusions share common psychophysical properties such as the relationship between the tilt angles of the stimuli and the evoked biases (Schwartz et al., 2007; Clifford, 2014). Our visual environment shares statistical redundancy as a common property across time and space. Under the efficient coding hypothesis, coding should be adjusted to reduce redundancy. Thus, both phenomena may be illusions related to the same redundancy-reduction process. Mechanisms involving adaptive gain control such as divisive normalization (Heeger, 1992) are speculated to be crucial for the tilt illusion and the tilt aftereffect. A more recent study suggested that the statistical similarity between the center and surrounding stimuli gates the surround suppression in the cortical neurons (Coen-Cagli et al., 2009, 2015). The following study revealed that short-term temporal regularities (e.g., stability of temporal input within one fixation) are learned and may explain the adaptation in the V1 neurons (Snow et al., 2017). However, no canonical model has ever simultaneously explained the multiple psychophysical phenomena, including tilt aftereffect and tilt illusion (Sanchez-Giraldo et al., 2019; Northoff and Mushiake, 2020).

The circuit responsible for surround modulation includes feedforward, feedback across areas, and lateral connections within an area (Angelucci et al., 2017). Feedforward connections contribute to a temporally fast and untuned component of surround modulation near the classical receptive field and emerge first in layer 4 in cats and primates in V1. Feedback connections contribute spatially extensive, and tuned components to surround modulation and are generated outside layer 4 (Angelucci et al., 2017). In V1, the target of feedback connection is both excitatory and inhibitory neurons (Gonchar and Burkhalter, 2003; Anderson and Martin, 2009). Horizontal connections are most prominent in layers 2/3 and contribute to a spatially extensive and tuned component and the modulation includes suppression and facilitation. The target of horizontal connections are excitatory and inhibitory neurons of similar orientation preferences at least in layers 2/3 (McGuire et al., 1991; Ko et al., 2011). To examine the effect of adaptation to the surround modulation, adaptation properties of the V1 neurons to simple artificial stimuli have been investigated. Multiple modulation patterns including enhancement, suppression, and change in tuning by adaptation have been observed (Wissig and Kohn, 2012; Patterson et al., 2013). One explanation for these multiple modulation types is the balance between the feedforward driving input, suppressive surrounding input, and divisive normalization (Dhruv et al., 2011; Solomon and Kohn, 2014). One of the speculated roles of these modulation types is to achieve a higher discriminability via the sharpening of tuning. However, the experimental results varied and were inconclusive, and even if sharpening was observed, whether it leads to an increase in discrimination accuracy by the neural populations remains unclear (Kohn, 2007; Cortes et al., 2012; Solomon and Kohn, 2014).

### 4.3 Gamma oscillations

Gamma frequency oscillations have been hypothesized to play a role in the relationship between the classical receptive field and the surround (Vinck and Bosman, 2016). In this section, I discuss gamma oscillations and adaptation. Gamma frequency oscillations are hypothesized to play a fundamental role in cortical processes such as attention (Fries et al., 2001; Fries, 2009). Another view is that gamma oscillations are reflections of the underlying cortical processing involving excitation-inhibition interactions and helpful indicators to detect such interactions (Ray and Maunsell, 2015; Bartoli et al., 2020; Ray, 2022). Reports have indicated that gamma oscillations increase in the supragranular layer of V1 neurons under repeated stimulus conditions (Hansen and Dragoi, 2011; Brunet et al., 2014). A study using laminar recordings and examining changes in the information flow due to stimulus repetition through current source density demonstrated that the effects of stimulus repetition (firing reduction) occurring in the supragranular layer due to adaptation subsequently propagated to other layers (Westerberg et al., 2019). This study suggests that the primary origin of repetition-related response modulation in V1 neurons is linked to intracortical processing within the supragranular layers. This does not necessarily exclude the inherited influences of suppression occur in retina. Some of the reduction in firing rate is likely due to a reduction in retinal input (Solomon et al., 2004). Thus, at least some of the modulation is speculated to originate from supragranular layers. In V1 neurons, the horizontal connections are prominent in layers 2/3 (Rockland and Lund, 1983; Lund et al., 1993; Angelucci et al., 2002) and I discussed that one of the circuit responsible for surround modulation is horizontal connections in the previous section. Horizontal connections are patchily distributed in the surrounding functional columns. These observations may suggest that interactions between neurons such as excitation-inhibition leading to gamma oscillations also relate to adaptation and occur primarily in layers 2/3.

As they progress to higher visual areas, gamma oscillations tend to decrease in power (Vinck and Bosman, 2016). The proportion of different inhibitory neuronal types differs across the visual areas (Kondo et al., 1994; Defelipe et al., 1999). Parvalbumin-GABAergic interneurons have been suggested to be involved in the generation of gamma oscillations and are abundant in layers 2/3 (Bartos et al., 2007; Tiesinga and Sejnowski, 2010). Furthermore, the lateral connections in layer 2/3 of the IT cortex exhibit a patchy pattern similar to those in the V1 cortex. However, unlike the V1 neurons, these connections do not decrease with distance, and the patch positions are more random in IT than in V1 (Fujita and Fujita, 1996; Tanigawa et al., 1998; Fujita, 2002). Thus, these differences in the connection patterns might explain the difference between the empirical gamma oscillation and the effect of adaptation across areas such as V1 (Hansen and Dragoi, 2011; Brunet et al., 2014), V4 (Wang et al., 2011), and IT (De Baene and Vogels, 2010). Generating gamma oscillations requires a considerable number of neurons for close synchronization. Synchronization of widely dispersed cells may be challenging to detect. While various insights into Gamma oscillation and adaptation in V1 are intriguing, anatomical circuit differences between V1 and IT may affect their experimental results and interpretation of RS in IT.

### 4.4 Adaptation in the lower visual area and RS in IT

In this section, I explored the existing hypotheses, experimental results, and implications obtained from the discussion of the adaptation in V1. This examination reveals a profound link between adaptation and context modulation, such as surround modulation. This possibly involves the interactions between inhibition and excitation from the observation of Gamma oscillation modulation in adaptation in V1 layer 2/3. As information processing transitions from V1 to IT, the represented information changes from the one strongly related to the outside environment, thus keeping the configuration of the input image to the one that is more behavior-relevant, abstract, and different from the input image. Thus, we do not have the strict equivalence of V1 surround modulation in IT. Still, modulation mechanisms that exhibit suppression and enhancement, possibly from lateral and feedback connections might be the important players in considering the mechanism of RS in IT.

As the adaptation occurs in the lower visual areas, the RS in the higher visual hierarchy of the IT may be influenced to some extent by the adaptation in the lower visual areas. The same stimuli were presented at non-overlapping locations to discriminate between inherited suppression and suppression occurring in IT (De Baene and Vogels, 2010). If adaptation in IT is inherited from earlier areas, adaptation would be absent when both the adapter and test stimuli do not fall within the same receptive fields at those earlier levels. The result showed suppression, indicating that suppression occurs within IT. However, the degree of suppression was less than at the same location, indicating some extent of suppression was inherited from earlier areas. Therefore, modulation occurs at each stage and is additive, with further modulation occurring in IT. Since modulation at each stage is dependent on the stimulus selectivity of the cells, the accumulated modulation in IT might be complex.

## 5 Predictive coding and RS

Predictive coding (Rao and Ballard, 1999; Friston, 2005; Spratling, 2008) is an influential proposal for visual processing that has evoked considerable discussion. In an original study (Rao and Ballard, 1999), a hierarchical neural network with neurons carrying prediction and error signals trained using natural images exhibited RF structures similar to those experimentally observed in the V1 neurons. This suggests that the network learns the statistical regularities of the natural images and conveys the signal deviations between the sensory inputs and such regularities (the error) to higher processing hierarchies to update prediction. This processing reduces redundancy by eliminating the predictability of the input signal, and thus, this process is consistent with efficient coding. Additionally, their model can be understood as a Bayesian framework of perception that assumes a generative model to infer the cause of the input. Several excellent and intuitive reviews on predictive coding are available (Kok and de Lange, 2015; Aitchison and Lengyel, 2017; Spratling, 2017; Keller and Mrsic-Flogel, 2018; Walsh et al., 2020; Shipp, 2024). This section will focus on RS and IT neurons in the context of predictive coding.

The most prominent characteristic of predictive coding is the presence of two neuronal types: neurons predicting sensory input and those coding prediction errors (the difference between predicted and observed sensory inputs) (Rao and Ballard, 1999; Friston, 2005; Bastos et al., 2012). The decrease in the response to repeated, thus redundant stimuli in the RS had been considered to be consistent with the behavior of error neurons (Auksztulewicz and Friston, 2016); however, closer examination speaks negatively about the presence of error neurons in the visual cortex of macaques, including the IT neurons (Kaliukhovich and Vogels, 2011; Vinken et al., 2018; Solomon et al., 2021).

In IT studies, following the experimental paradigm of Summerfield et al. (2008) in which alterations in stimulus identity were expected in some blocks and repeats were expected in others, neuronal responses to the stimuli in the expected and unexpected condition were compared (i.e., alterations in repeat blocks or vice versa). The results demonstrated modulation dependent on the repeats, which is presumably RS, but did not show modulation due to expectation violations (Kaliukhovich and Vogels, 2011). However, if the animal was exposed to the fixed ordered stimuli for an extended period (they controlled the probability of the order of the stimulus), the neural response to the stimuli of expected order was shown to decrease (Meyer and Olson, 2011; Meyer et al., 2014; Kaposvari et al., 2018). Such a suppression caused by expectation is called expectation suppression (ES). These studies revealed that ES was observed when the animal is under some belief situations (they believe that a particular stimulus comes after a corresponding particular stimulus); however, RS was observed regardless of the belief, suggesting that different neural mechanisms drive the two phenomena (Vogels, 2016; Vinken et al., 2018). RS is suggested to be the result of relatively low-level automatic processes (Feuerriegel et al., 2021), which differs from ES.

Is RS in IT a process distinct from predictive coding? If predictive coding is the fundamental principle of visual computation, why can it not explain phenomena such as RS, which are broadly observed in the ventral visual area, regardless of additional constraints such as attention, awareness, or exposure time? Keller and Mrsic-Flogel (2018) highlighted the challenges in controlling the expectations of animals or systems and emphasized that researchers can access them at best through certain proxies for them. We lack direct access to elements such as “expectations” and “errors” within the visual hierarchy. It is highly likely that the neural correlate of these concepts is obscured in the complex visual system and not straightforward to comprehend. Another possibility, as highlighted by Cao (2020), is that the information processing structure might not essentially differ from the traditional scheme, where stimulus representation ascends across the hierarchy and is integrated through feedforward adjusted by feedback (Heeger, 2017), thus we may not need to choose or deny either. Some predictive coding models are proposed that do not explicitly assume error neurons (Spratling, 2008; Sihn and Kim, 2022). The evaluation of these models is intriguing. Without identifying specific circuits, the appropriate explanation (Shipp, 2024) or whether they essentially express the same concept remains uncertain.

## 6 RS in fMRI study

In fMRI studies of visual object perception and recognition, research has been conducted on RS and adaptation from several different perspectives. In this section, I discuss fMRI studies focusing on the property and mechanism of RS and adaptation in higher ventral visual areas such as fusiform face areas (FFA).

fMRI adaptation has been used to investigate area selectivity (Grill-Spector and Malach, 2001; Malach, 2012). The logic of the experiments is that the adaptation causes weakened responses to repeated or prolonged stimuli. If altering the properties of a stimulus causes fMRI responses to recover, this is evidence that a distinct population of neurons has been recruited by the stimulus manipulation. Equivalently, stimulus-specific adaptation effects on fMRI responses indicate the presence of neurons that are selective along the dimension of stimulus manipulation (Larsson et al., 2016). However, there is criticism that the fMRI method, which captures mass activity as changes in BOLD signals, makes it difficult to accurately infer the modulation of neuronal activity that varies in many ways, including increased and decreased activity, fatigue, sharpened tuning, response facilitation, and altered response dynamics (Larsson et al., 2016). In addition, slow response obscures the difference between changes within the area and those coming from other areas.

In other studies, a relationship between Predictive Coding and RS has been investigated. Summerfield et al. (2008) demonstrated that the BOLD signal in FFA is greater for unexpected face stimuli compared to expected face stimuli, suggesting that RS is related to expectation rather than adaptation, thereby supporting predictive coding. However, this type of expectation-related modulation requires attention, while RS occurs even in the absence of attention (Larsson and Smith, 2012). Additionally, electrophysiology studies in monkey IT have shown that neural activity modulation related to expectation is not observed with such short exposures (Kaliukhovich and Vogels, 2011), providing negative evidence against this interpretation. However, by analyzing RS from the perspective of how inter-area relationships change, Ewbank et al. (2013) demonstrated a connection with predictive coding. They recorded the BOLD responses of the FFA and OFA to face stimuli of the same or different sizes. They applied dynamic causal modeling to examine the effects of stimulus repetition. They reported that the repetition of the same face was associated with changes in forward (OFA-to-FFA) connectivity. In contrast, the repetition of a face of a different size was characterized by altered backward connectivity (FFA-to-OFA), insisting the finding is consistent with predictive coding. Another study showed that inter-regional (ACC-FFA) coupling of BOLD signal increases by stimulus repetition, indicating another explanation of the mechanism of RS, based on synchrony (Gotts et al., 2021).

## 7 Other computational implications for RS

So far, I have explored the neural processes and anatomical structures related to RS, drawing insights from lower visual areas. The RS appears to be intimately connected to the computations in the visual areas. Herein, I further delve into the potential significance of RS in processing visual information in the brain. The models I discuss in this section provide insights into RS. I discuss models that explore the dynamics of neural networks in response to changing inputs, focusing on the explanation of RS, or, in a broader sense, on the visual environment dynamics. The first model examines how neural networks with selective inhibitory connections can achieve inference-based visual processing, which explicitly uses prior and posterior probability, and explains RS (Lochmann et al., 2012; Chalk et al., 2018). The second model incorporates hierarchical processing while integrating efficient coding principles (Snow et al., 2017, Młynarski and Hermundstad, 2018, Park and Pillow, 2024). The final discussion includes spiking neural networks that maintain a tight E/I balance and coding stability (Denève et al., 2017; Gutierrez and Denève, 2021). This network model shows how low energy, thus high-efficiency constraint, can achieve stable population coding.

### 7.1 Inhibitory connections

Numerous models that incorporate divisive normalization (Heeger, 1992) have been proposed given the wealth of experimental findings suggesting that divisive normalization forms the basis of rich contextual modulation (Northoff and Mushiake, 2020). Among them, Lochmann et al.’s (2012) model distinguishes itself from other models for natural RS reproduction. Their spiking neural network model included feed-forward and competitive inhibitory lateral connections. In their study, the network was a generative model that inferred the probability of the existence of an object (or feature) in a visual image, and the inhibitory connections made the inference of different objects competitive. In repeated-stimulus conditions, the probability of the first object (adaptor stimulus) remains high in the period immediately following its disappearance because the probabilities are updated by the slow integration of the sensory input. This explains why the input of repeated stimuli results in a substantial reduction in the detector gain for that stimulus. Their model is not strictly mapped to real neural circuits but is intriguing in that it explains contextual modulation and RS not as a model describing the phenomenon itself, but as an ingredient for realizing the essential purpose of visual processing, which is the inference of the visual world. A subsequent study (Chalk et al., 2018) demonstrated noise-invariant output in the network that had input-targeted divisive inhibition but less constraint on the stimulus and their neural code than the previous one. Another group showed that the network model trained by natural movies learned the statistics of the movie, which exhibited a response similar to adaptation in V1 via divisive normalization over time (Snow et al., 2017). This model includes a modulation mechanism similar to the V1 surround modulation with divisive normalization, which functions as a statistical inference of the classical receptive field using the surrounding information (Coen-Cagli et al., 2015). The model divisively normalizes the present visual input using past visual inputs only to the extent that they are inferred to be statistically dependent. These models explain the role of inhibitory inputs in visual processing and suggest their potential to simultaneously account for RS.

### 7.2 Multiple aspects of efficiency

High-fidelity encodings in which precise reconstruction of coded information is possible can be metabolically costly, but low-fidelity encodings can lead to errors in inference. The visual system may experience a tradeoff between these two efficiencies. A model based on input statistics was proposed to balance this tradeoff (Młynarski and Hermundstad, 2018). This model aims to maintain an accurate estimate at a minimal cost. Specifically, the current state (stimulus distribution) is estimated from the response as a firing rate, and this estimation, when fed back into the encoding scheme, adjusts the coding fidelity. In other words, the coding fidelity and metabolic costs are prioritized in situations with high uncertainty and certainty, respectively. A Bayesian observer was used to implement the model. They assumed a visual hierarchy as an encoder and observer being the V1 and V2 neurons, respectively, and demonstrated changes in the firing rates depending on the two scenarios. Bursts occur when the statistical distribution of the stimulus (which is assumed to be coded by the V2 neurons) becomes more uncertain, resembling responses to breaking statistical regularities. Conversely, bursts did not occur when the stimulus distribution was similar to the previous one, qualitatively aligning with response suppression.

Park and Pillow (2024) proposed a framework that combines Bayesian inference and efficient coding (efficient Bayesian coding). The framework includes prior distribution, encoding model, capacity constraint, and loss function, making it possible to compare different types of loss function. The study demonstrates multiple cases suggesting that the original efficient coding (information maximization) may not be relevant. Although these models do not directly predict the underlying computation and a clear connection with the roles of different cells in specific circuits of the visual cortex, the perspective of optimizing multiple objectives may be crucial for comprehensively understanding the visual system and adaptation.

### 7.3 E/I balance

The final topic is the E/I-balanced network. Excitation and inhibition have been shown to be tightly balanced in the brain (ex. Xue et al., 2014). In the network model of Gutierrez and Denève (2021), the adaptation of the spike-frequency and E/I-balanced recurrent connectivity have emerged as solutions to the global cost-accuracy tradeoff. This network redistributes the sensory responses from highly excitable to less excitable neurons as the cost of neural activity increases. This change does not alter representation at the population level, despite dynamic changes in the individual neurons (Gutierrez and Denève, 2021). The idea of a trade-off between metabolic cost and coding accuracy is similar to that of the aforementioned models. This model is unique in that the conflict is solved using the circuit property of the E/I balancing network, which is a characteristic of the brain. In this model, an autoencoder is used, and the optimization of the coding accuracy involves directly representing the input as the output. Investigating how the hierarchy of visual processing and changes in coding are expressed within this context would be intriguing.

## 8 Discussion

RS or adaptation has been extensively investigated for a long time, and its significance has been debated across various perspectives. Surprisingly, there are still aspects that remain unclear. Considering the widespread observation of adaptation in visual processing, it is speculated to have deep implications for its computational principles of visual systems. Therefore, elucidating adaptation and RS might be intertwined with understanding the visual computational principles. Insights from electrophysiological studies, anatomical observations, and modeling need to be integrated for a comprehensive understanding beyond what is currently available.

Several studies showed that neurons in lower visual areas efficiently code visual stimuli by maximizing the information that can convey natural image inputs. In contrast, in middle and higher visual areas, optimization may not be for the efficient representation of the input image but for the representation of the output, i.e., various behaviors that can be realized. In the case of the IT cortex, which is closest to the output for behavior, this optimization may strongly depend on the behavior.

The computational principle of the visual system depends on the unique features of the visual system including two-dimensional spread, and the temporal dynamics of input resulting from frequent saccades and fixations. These factors may contribute to the specificity of the visual system, and fundamental commonalities with other modalities are possibly concealed. In recent years, research using optogenetics in rodents has expanded, providing valuable insights. While some findings may be translated, cautious interpretation is essential due to potential differences in the hierarchical structure and anatomical circuit between primates and rodents. Massive and hopefully comprehensive recordings of population activity are necessary to unveil the underlying principles.

In summary, IT neurons dynamically adapt their responses to visual stimuli based on experience, showing robust RS. As a neural activity modulation in which responses change upon repetition of the same stimulus, RS and adaptation is prevalent throughout the visual system and transcends hierarchical levels. RS in IT cannot be explained simply by fatigue as it selectively occurs in response to specific stimuli; instead, it is considered a phenomenon related to the fundamental aspects of visual processing. Insights from studies on similar phenomena in lower-order processing help speculate RS in IT neurons. Adaptation involves inhibitory contextual modulation, which may be related to gamma synchronization. Explaining RS from the efficient or predictive coding perspective may be possible; nevertheless, evaluating efficiency is not straightforward and must be carefully considered. Multiple types of efficiency including metabolic efficiency, read-out efficiency, or coding efficiency can contribute simultaneously to the visual system. In addition, contributions can differ in the visual processing stages. The inhibitory neurons as well as excitatory and inhibitory balance may be critical players in adaptation. RS is an intriguing subject for understanding visual processing and connecting various hypotheses and psychological phenomena, and comparisons across different visual hierarchies and between different methods are essential to understanding its mechanism and role.

## Author contributions

YY: Writing – original draft, Writing – review & editing.
